# Synthesis and Optimization of Cs_2_B′B″X_6_ Double Perovskite for Efficient and Sustainable Solar Cells

**DOI:** 10.3390/molecules28186601

**Published:** 2023-09-13

**Authors:** Ruijia Yao, Tingxue Zhou, Shilei Ji, Wei Liu, Xing’ao Li

**Affiliations:** 1 New Energy Technology Engineering Laboratory of Jiangsu Province, Institute of Advanced Materials, School of Science, Nanjing University of Posts and Telecommunications (NJUPT), Nanjing 210023, China; 2 School of Science, Zhejiang University of Science and Technology (ZUST), Hangzhou 310023, China

**Keywords:** lead-free double perovskite, synthetic approaches, solar cells, long-term stability, crystallization, band gap adjustment

## Abstract

Hybrid perovskite materials with high light absorption coefficients, long diffusion lengths, and high mobility have attracted much attention, but their commercial development has been seriously hindered by two major problems: instability and lead toxicity. This has led to lead-free halide double perovskite becoming a prominent competitor in the photovoltaic field. For lead-free double perovskites, Pb^2+^ can be heterovalent, substituted by non-toxic metal cations as a double perovskite structure, which promotes the flexibility of the composition. However, the four component elements and low solubility in the solvent result in synthesis difficulties and phase impurity problems. And material phase purity and film quality are closely related to the number of defects, which can limit the photoelectric performance of solar cells. Therefore, based on this point, we summarize the synthesis methods of Cs_2_B′B″X6 double perovskite crystals and thin films. Moreover, in the application of solar cells, the existing research mainly focuses on the formation process of thin films, band gap adjustment, and surface engineering to improve the quality of films and optimize the performance of devices. Finally, we propose that Cs_2_B′B″X_6_ lead-free perovskites offer a promising pathway toward developing highly efficient and stable perovskite solar cells.

## 1. Introduction

In response to the problem of climate change caused by the burning of fossil fuels, the development of renewable-energy-generation materials has inspired a lot of research. Metal halide perovskites have sparked enormous research interest due to their adjustable band gap, long carrier diffusion length, high carrier mobility, and convenient manufacturing processes, which inspire hope for third-generation photovoltaic materials. In 2009, hybrid perovskite solar cells (PSCs) were prepared by Miyasaka et al. with a power conversion efficiency (PCE) of 3.8%, which is now certified up to 26.1% [1]. However, two serious problems have prevented the commercial application of hybrid perovskite solar cells. One is that hybrid perovskite is unstable under humidity, heating, and light [2]. The other is the toxicity of hybrid perovskite, which has led researchers to search for elements that could replace it [3]. Fortunately, researchers have used monovalent and trivalent positive ions, instead of lead, to form stable double perovskite structures, which has solved both the toxicity and stability problems [4,5,6]. For example, the hotspot material is Cs_2_AgBiBr_6_; in addition to that, there are Cs_2_AgSbBr_6_, Cs_2_AgInCl_6_, Cs_2_CuBiBr_6_, Cs_2_NaBiCl_6_, and so on [7]. Nevertheless, compared with hybrid perovskites (such as MAPbI_3_), Cs_2_B′B″X_6_ perovskites have one more type of element, which leads to complexity in material synthesis [8,9,10]. It is necessary and interesting to investigate lead-free double perovskite materials and their applications in solar cells.

There are several reasons why the preparation process of material synthesis and film formation still needs a lot of research. (1) The solubility of double perovskite precursors in organic solvents is low; (2) the conditions for pure-phase films are strict; and (3) the film preparation process has not been perfected. Moreover, the unique synthetic chemistry makes the quality of the double perovskite film largely dependent on the deposition process, which has a significant impact on the device’s performance [11]. Therefore, it is very important to prepare pure double perovskite materials efficiently. For better synthesis of crystalline materials, scientists have proposed using the solid-phase method to synthesize pure-phase double perovskite, and many new ideas about temperature control based on solution synthesis have been put forward [12]. With the intensification of research, scientists have directly dissolved the material powder in the solvent at a certain temperature and speed in order to complete the experiment, forming the precursor solution [13], which has also greatly reduced the cost and increased convenience. At the same time, in addition to crystal synthesis, thin films are an additional important consideration for solar cells. A thin film is a form of the large-area presentation of crystals, which is usually made via vapor deposition and solution deposition. Nowadays, the thin films of PSCs have been fabricated by numerous research groups and companies, using approaches to improve coverage such as screen printing, inkjet printing, slot die printing, spray coating, and doctor blading [14,15,16]. Different preparation methods have different characteristics. For example, Wang et al. successfully developed Cs_2_AgBiBr_6_ perovskite film with dense particles prepared via the vapor deposition method, and the defect density (2.13 × 10^16^ cm^−3^) was lower than that of the solution method (9.1 × 10^16^ cm^−3^) [17]. The methods of crystal synthesis and film formation have gradually been improved and diversified.

As is widely known, pure-phase materials and complete films are the basis of perovskite solar cells, but in the preparation of solar cells, defects in the block materials and a reduction in surface defects are always problems. Therefore, more methods and strategies are needed to further improve the Cs_2_B′B″X_6_ double perovskite solar cells [18,19,20]. First, performance can be optimized by improving the film production process. For example, in the film-manufacturing process, an anti-solvent was added to the spinning and coating method, which effectively improved the smoothness and crystallinity of the thin film. Gao et al. achieved excellent results when they spun and coated the Cs_2_AgBiBr_6_ film with appropriate solvent resistance technology, which significantly reduced the defect density of perovskite film and increased the PCE to 2.2% [21]. Secondly, performance can also be improved by band gap adjustment. Generally, structural transitions and functional doping can be utilized to adjust the band gap. The structural characteristics of double perovskite, with a large indirect band gap and poor absorption capacity, lead to its poor applicability in photovoltaic devices [22]. Like Cs_2_AgBiX_6_, the antibonding states of Ag 5s and Bi 6p with halogen 3p/4p are involved in the transition from conductance to the valence band, and this interaction results in an indirect band gap [23,24]. However, through structural changes or chemical composition changes, the band gap can be reduced or even directly changed into a direct band gap, thus improving the photoelectric performance of the device [25,26,27]. For example, Li et al. successfully reduced the band gap of single-crystal Cs_2_AgBiBr_6_ by applying a high pressure of 15 GPa, but the crystal structure and band gap recovered rapidly with the removal of the pressure [28]. Recently, Ji et al. changed the band gap from 1.98 eV to 1.72 eV in Cs_2_AgBiBr_6_ single crystals by adjusting the Ag-Bi disorder [29]. Thirdly, further improvements can be made using surface-engineering methods such as band gap alignment and surface passivation. When carriers are transported from the light-absorbing layer to the transport layer, it is first necessary to ensure that the band between the two layers is matched in order to effectively transport electrons or holes. At the same time, carrier localization may occur due to surface defects during interface transport, resulting in carrier recombination. Nowadays, using organic molecules as passivating agents for the repair of surface defects is a good choice. Therefore, it is of great significance to explore the effects of film optimization, band gap adjustment, and interface improvement on solar cells in order to accelerate the exploration of the synthesis and improvement strategies of Cs_2_B′B″X_6_ double perovskite solar cells. In this review, the material synthesis and thin-film preparation of Cs_2_B′B″X_6_ double perovskite are introduced. Among them, the material synthesis is mainly divided into solid-state synthesis and solution-based synthesis, and vacuum deposition and chemical solution deposition have become the main ways to manufacture thin films. In the context of successful synthetic materials, the performance of perovskite solar cells can be enhanced through film quality optimization, band gap adjustment, and interface engineering strategies. Finally, the challenges of double perovskite are summarized, the assumption that lead-free halide double perovskite compounds can be used as effective substitutes for lead-based perovskite is prospected, and the possible solutions are proposed.

## 2. Crystal Synthesis of Cs_2_B′B″X_6_ Double Perovskite

**Figure 1:**
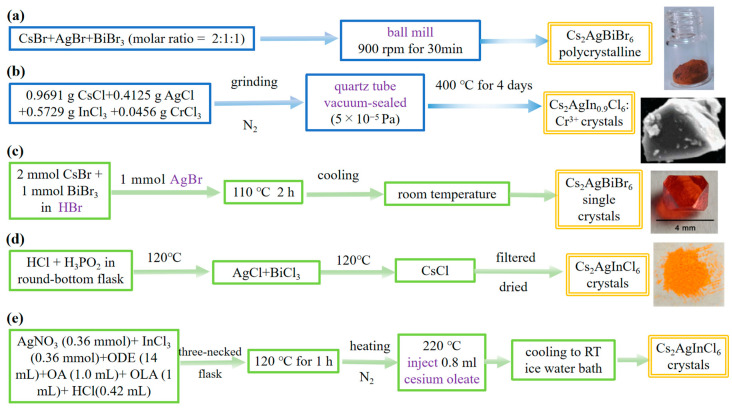
Flow chart for synthesis of lead-free halide double perovskite crystals by solid-phase reaction (**a**,**b**) ((**a**) Reproduced with permission [47], 2019, Royal Society of Chemistry. (**b**) Reproduced with permission [41], 2019, Royal Society of Chemistry.), and solution treatment (**c**–**e**) ((**c**) Reproduced with permission [36], 2016, American Chemical Society. (**d**) Reproduced with permission [34], 2016, American Chemical Society).

In the current research progress, Cs_2_AgBiBr_6_ with its high light absorption coefficient, good stability, and environmental friendliness is the most popular among the lead-free double perovskites with Cs_2_B′B″X_6_ configuration. It can be applied in a variety of photovoltaic devices, such as solar cells, photocatalysis, sensors, and so on. And other materials like Cs_2_AgBiCl_6_ single crystals with high sensitivity can be used in UV detectors [30]. Therefore, it is crucial to synthesize pure crystals. Compared with the common ABX_3_ hybrid perovskite, the perovskite elements are more diversified, so the synthesis method will be more complex, which means that it has higher requirements for the synthesis of crystals [31,32,33]. Table 1 shows the summary of crystal synthesis (solid-state synthesis/solution synthesis) in recent years. To be specific, crystal synthesis can be divided into solid-phase synthesis and solution synthesis. Solid-state synthesis means that crystals are synthesized directly using instruments without adding solvents, but solution synthesis is any synthesis in the presence of a precursor solution, and eventually, some or all of the elements are dispersed and dissolved in the solvent. Therefore, Slavney, McClure, and Volonakis et al. started the preparation of halide double perovskite at almost the same time, proposed the method of double perovskite synthesis, and then summarized it [34,35,36]. Moreover, for Cs_2_B′B″X_6_ double perovskite, solid-state synthesis mainly included the ball mill method and high-temperature solid-phase synthesis. Solution-based synthesis is more diverse, like the hydrothermal method, the precipitation method, the hot injection method, the spray-drying method, and so on (Figure 1).

**Table 1:** Summary of synthesis conditions of Cs_2_B′B″X_6_ double perovskite compounds.

Compound	Synthesis Method	Synthesis Atmosphere	Heating Condition	Year and Reference
Cs_2_AgInCl_6_:Cr^3+^	hydrothermal		150 °C for 12 h	2022 [37]
Cs_2_AgInCl_6_:Cu^2+^	hot injection	N_2_	heating at 120 °C for 1 h, 220 °C for injection	2020 [38]
Cs_2_AgInCl_6_:Mn^2+^	hot injection	vacuum and N_2_	dry under vacuum for 30 min at 40 °C, 105 °C for injection	2018 [39]
Cs_2_AgInCl_6_	hot injection	vacuum and N_2_	100 °C in vacuum for 2 h, 200 °C in N_2_ for injection	2019 [40]
Cs_2_AgInCl_6_:Cr^3+^	solid-state reaction	evacuated ampoules	400 °C for 4 days	2019 [41]
Cs_2_AgInCl_6_	hot injection		100 °C for injection	2019 [42]
Cs_2_AgInCl_6_:Mn^2+^	solution processing		72 °C for 20 min	2018 [43]
Cs_2_AgInCl_6_:Yb^3+^	hot injection	vacuum and N_2_	100 °C in vacuum for 15 min, 105 °C in N_2_ for injection	2019 [44]
Cs_2_Ag(Sb_x_Bi_1−x_)Br_6_	solution processing	N_2_	RT	2020 [26]
Cs_2_AgSbBr_6_	hot injection	vacuum and N_2_	110 °C in vacuum for 45 min, 180 °C in N_2_ for injection	2018 [45]
Cs_2_AgSbBr_6_	hydrothermal		160 °C for 5 days	2019 [46]
Cs_2_AgSbBr_6_	solid-state reaction		high-energy ball mill	2019 [47]
Cs_2_AgSbBr_6_	solid-state reaction		planetary ball mill	2022 [48]
Cs_2_AgSbBr_6_	solution processing		383 K	2022 [48]
Cs_2_AgSbBr_6_	hydrothermal	N_2_	heat up to 473 K in 10 min with a pressure of 4.7 MPa	2022 [48]
Cs_2_AgSbBr_6_	solid-state reaction	inert loop system	spray-drying for 474 K	2022 [48]
Cs_2_AgBiBr_6_	solution processing		120 °C for 3 h	2021 [49]
Cs_2_AgBiBr_6_	solution processing		100 °C	2018 [50]
Cs_2_AgBiBr_6_	hydrothermal		120 °C for 24 h	2020 [51]
Cs_2_AgBiBr_6_	solution processing		110 °C for 2 h	2017 [52]
Cs_2_AgBiBr_6_	solution processing		RT for 2 h	2021 [53]

### 2.1. Solid-State Synthesis

Solid-state synthesis is the coupling of mechanical and chemical energy, allowing precursors to mix and grind to form chemical reactions. As a solvent-free method, the desired double perovskite can be formed and the reaction time can be greatly reduced.

The synthesis of Cs_2_AgSbBr_6_ by a high-energy ball mill is reported by Garcia-Espejo et al. in Figure 1a. Polycrystalline powders were formed by mixing CsBr, AgBr, and SbBr3 at a molar ratio of 2:1:1. The ball mill uses two 125 mL reaction chambers, one of which is placed with fourteen 10 mm stainless steel balls, and the precursor mass of each chamber is about 1–2 g. The high-energy ball mill lasts at 900 rpm for 30 min and finally forms the double perovskite phase. This is caused by mechanical forces to chemically induce the formation of new phases by the three bromides, which generate intrinsic heat during the ball milling process. The authors have also successfully synthesized pure-phase Cs_2_AgBiBr_6_ and MA_2_TlBiBr_6_ materials. But for synthetic Cs_2_AgSbBr_6_, despite the presence of the Cs_3_Sb_2_Br_9_ side phase, this is a big step forward on the road to successful Cs_2_AgSbBr_6_ synthesis. When stored at room temperature and ambient humidity for several months, the lattice structure does not change. This mechanochemical method is a new approach that requires no solvent and no heating, and it is economical, environmentally friendly, and fast. However, the mixture-phase problem of some materials needs to be further explored. Due to the presence of decomposition reactions, this will pave the way for the preparation of other thermodynamically unstable double perovskites [47].

Moreover, another solid method is the solid-phase sintering method, which can effectively obtain the crystal. Zhao et al. used high-temperature solid-phase synthesis of Cs_2_AgInCl_6_ doped with Cr in Figure 1b. The solid-phase sintering method is a traditional and environmentally friendly method to generate a new phase with the characteristics of a high temperature and vacuum environment. However, it can be obviously found that these temperature requirements are high and time-consuming, which is weak in practical applications. The ground mixture was vacuum-sealed (5 × 10^−5^ Pa) in a quartz tube, calcined in a box furnace at 400 °C for 4 days, and cooled to room temperature to form the desired sample. However, in the process of solid-state reaction at a high temperature, it produces secondary phases of Cs_3_InCl_6_ and CsAgCl_2_. And because of doping, not all Cr^3+^ ions enter into the lattice, and the segregation phase of CrCl_3_ will be formed so that the actual content of Cr^3+^ is smaller than that in the standard composition [41]. Moreover, the impurity phase cannot be reduced by second-round sintering or at a lower temperature method. Other phase materials have a negligible impact. This also provides good guidance on material synthesis. 

### 2.2. Solution-Based Synthesis 

Solution-based synthesis is different from solid-state synthesis because solid-state synthesis is instrument-dependent, while solution synthesis is cheaper and more convenient. The hydrothermal method is the process of crystallization under pressure heating, which can form high-purity and well-crystallized materials. However, this method is not suitable for water-sensitive materials. But the solvothermal method, which is similar to the hydrothermal method, makes up for this shortcoming. In 2016, Slavney et al. used the HBr route to successfully synthesize Cs_2_AgBiBr_6_ (Figure 1c) [36]. With the deepening of research, Chen et al. dissolved 2 mmol of CsCl, 1 mmol of InCl_3_, and 1 mmol of AgCl in 10 M HCl, placed the solution in a 50 cm^3^ high-pressure reactor, heated it at 150 °C for 12 h, and then cooled it to 60 °C at a cooling rate of 3 °C h^−1^. The Cs_2_AgInCl_6_ crystals were filtered, washed with isopropanol, and dried in an oven at 60 °C for 3 h [37]. It is worth noting that there is no noticeable impurity produced. And Cr^3+^ has also succeeded in partially replacing In^3+^. Compared with the solid-phase sintering method, the hydrothermal method can obtain more pure crystals.

There is another solution-based method to synthesize Cs_2_B′B″X_6_ double perovskite crystals, which is called the precipitation method. With the help of a solvent, the halide is dissolved and crystallized by heating. This method is simple and convenient to operate, but the requirement of material morphology is not high. McClure et al. prepared Cs_2_AgBiX_6_ samples by precipitation in hydrohaloic acid and hypophosphoric acid solutions [34]. The HCl and H_3_PO_2_ solution was added to the round-bottomed flask and heated to 120 °C (Figure 1d). AgCl and BiCl_3_ were added first, and then CsCl was added to the hot solution. The precipitate was removed, washed with ethanol, and placed to dry. On the other hand, through solid-state synthesis, the author ground CsCl, AgCl, and BiCl_3_ at a molar ratio of 2:1:1 for 20 min, and then put the powder in an alumina crucible, placed it in a box furnace, and heated it in air at 210 °C for 10 h, after two thermal cycles, to form the required sample. In addition, the same solid-state synthesis of Cs_2_AgBiBr_6_ and Cs_2_AgBiCl_6_ was performed. Cs_2_AgBiBr_6_ can approach a pure phase until heated to 210 °C. But for Cs_2_AgBiCl_6_, BiCl_3_ absorbs water, which results in the replacement of the double perovskite phase. Moreover, comparing the solid method and solution-based method, the samples synthesized by the solid-phase method have secondary phase production, while the solution-based method can make higher-purity materials. 

The hot injection method is very common in chemical synthesis, and it is no exception in perovskite synthesis. It means that the precursor solution is injected into the reaction solution at a certain temperature to make rapid crystal nucleation; this method is easy to control and can obtain a good purity product, but the required temperature is high. Lee et al. suspended Cs_2_CO_3_, silver acetate (Ag(OAc)), and indium(III) acetate (In(OAc)_3_) in a mixture of 1-octadecene (ODE), oleic acid (OA), and Oleylamine (OAm) and degassed at 100 °C for 2 h (Figure 1e). Then, the temperature was increased to 200 °C under N_2_ atmosphere, and Chlorotrimethylsilane (TMS-Cl) in 1.13 mL of ODE was rapidly injected. After centrifugation, dispersion, and so on, the crystal was finally obtained [38]. Although it needs a high temperature, no secondary phase was found by X-ray Diffraction (XRD) and Energy Dispersive Spectrometer (EDS) characterization.

There are many methods for the synthesis of lead-free double perovskite materials, and each method has its own characteristics. As for the new materials formed by the doping of elements in Cs_2_B′B″X_6_ double perovskites, the results presented by different synthesis processes are not the same because of more material elements. Yoon et al. studied five synthetic methods for using Sb to replace Bi in Cs_2_AgBiBr_6_ [48]. They are mechanochemistry, the solution cooling method, the microwave-assisted hydrothermal method, the HBr route, and spray-drying. Respectively, the spray-drying method is a special method. CsBr, BiBr_3_, and AgBr were dissolved in dimethyl sulfoxide (DMSO). The solution was stirred overnight at room temperature. Subsequently, the precursor solution was spray-dried using a B-290 mini-spray dryer to form crystals.

For the mechanical method, no more than 40% of the doped Sb exists in the pure phase. It has been theorized that when the ball generates a temperature under collision, this leads to local heating, and the molecular diffusion rate increases, promoting phase formation. But also because the element is insoluble, it can only be partially doped. For the solution cooling method, the microwave-assisted hydrothermal method, and the HBr route, any proportion of the doping will produce a secondary phase. While SbBr_3_ is easy to decompose in water, resulting in the formation of secondary phases in HBr route method. It is worth noting that spray-drying is a fast process where the solution is sprayed and the quenching effect is dominant. Moreover, DMSO is used as a solvent, and the dissolution effect is better, but it is also because of the rapid process, more defects may be generated, so the doping concentration is limited. Therefore, up to 40% of Sb substitution in Cs_2_AgBiBr_6_ can be achieved by solid-state synthesis and spray-drying methods, and both methods have their own characteristics. But the other three methods hardly replaced Bi in Cs_2_AgBiBr_6_. 

Many synthetic material methods have been studied, but for the mass production of powder, the spray-drying method is carried out. The spray-drying method mainly converts liquids into solids to form powder [54]. The feed liquid is atomized using a nozzle to form small droplets, which are then put in contact with the hot carrier gas to promote solvent evaporation, thus forming particles. Mainly, when the solubility of the solution reaches its limit, the particles will precipitate or crystallize. This method was first applied to the Cs_2_AgBiBr_6_ material, and the obtained powder has good crystallinity and no secondary phase. Compared with other traditional materials, the powder quality is the same, but the production efficiency will be higher. This innovative approach could pave the way for the synthesis of other materials and also enable the mass synthesis of materials.

## 3. Thin-Film Preparation of Cs_2_B′B″X_6_ Double Perovskite

In the solar cell absorption layer, not only should the synthesized material phase be as pure as possible, but the film quality is also critical. The quality of thin-film formation will directly affect the performance of the device. For example, if there are too many visible defects in the film during the preparation of the film, the device filling factor is not ideal, and obviously, it cannot show excellent photoelectric performance [55,56,57]. So far, halide lead-free double perovskite films can be roughly divided into two types, respectively, which are vapor deposition and chemical solution deposition. Table 2 exhibits thin-film synthesis (vapor deposition/solution deposition) in recent years. Especially, chemical solution deposition is more popular because of the cost and ease of processing.

### 3.1. Chemical Solution Deposition 

Chemical solution deposition is a method of depositing thin films on the substrate surface by chemical reaction or electrochemical reaction in the process of film preparation. Solution deposition can accurately control the stoichiometric ratio, it has good uniformity, low costs, high material utilization, and low energy consumption, but it can also synthesize material particle size and shape, and the orientation can be better controlled [62]. In the preparation of hybrid perovskite films, the process is more mature. The common methods are mainly the one-step method, the two-step method, the soaking method, the scraping method, and so on [63]. For A_2_B′B″X_6_ materials, due to the diversity and solubility, the main film preparation process is the one-step method, and the other methods need to be continuously explored. In this section, interesting methods for forming thin films are introduced.

**Figure 2:**
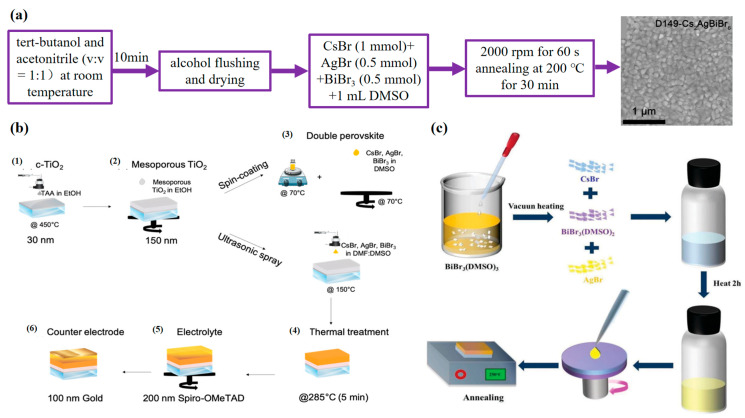
Double perovskite thin films synthesized via solution deposition. (**a**) Flow chart of chemical solution deposition of Cs_2_AgBiBr_6_ doped with D149 (reproduced with permission [65], 2021, American Chemical Society); (**b**) overview of the manufacturing process of the Cs_2_AgBiBr_6_-based solar cells (reproduced with permission [61], 2021, John Wiley and Sons); (**c**) schematic diagram of the DMSO adduct approach to deposit the perovskite (reproduced with permission [53], 2021, Royal Society of Chemistry).

In 2017, the Cs_2_AgBiBr_6_ film was first made and incorporated into the working device [64]. Specifically, the preheated precursor solution was spun onto the electron transport layer at 2000 rpm and annealed at 285 °C for 5 min to form a pure-phase film. Through the successful experience of previous workers, many researchers continue to study the preparation of thin films. Wang et al. mixed CsBr, AgBr, and BiBr_3_ in a DMSO solution to prepare the perovskite precursor solution (Figure 2a). The precursor solution was uniformly coated on the electron transport layer by the one-step spin-coating method, and finally, a good film shape was formed after annealing [65]. In addition, the indole dye was soaked and dried to form a film. This improves the crystallization process of Cs_2_AgBiBr_6_, resulting in a dense light-absorbing layer, and also facilitates electron transport between the electron transport layer and the light-absorbing layer. The one-step process has the advantages of low cost and simple operation, and it has become the most widely used film-forming technology at present. For the soaking method and the two-step method, the process is complex, and the process factors can be accurately grasped to obtain a well-crystallized film.

Ultrasonic spray deposition is a scalable, semi-automatic atmospheric pressure process. As shown in Figure 2b, Daem et al. prepared 0.15 M of the Cs_2_AgBiBr_6_ precursor solution by mixing BiBr_3_, AgBr, and CsBr in N, N-dimethylformamide (DMF)/DMSO (volume ratio = 4:1). It is important to note that the precursor solution concentration is reduced in the spraying method, and the spray deposition ratio is determined to reduce material loss. The precursor solution is stirred overnight and ultrasonically sprayed with a matching nozzle and system to obtain a smooth and uniform film after annealing. And the thickness of the film has no pinhole, and the grain is compact and flat [61]. In addition, ultrasonic jet deposition can optimize the light absorption and charge the transfer between the Cs_2_AgBiBr_6_ and the hole transport layer, which effectively improves the photoelectric performance of the entire device. From the comparison between the spraying method and the one-step method, it can be seen that although good equipment is needed, the spraying method is a good choice.

As shown in Figure 2c, Hou et al. make adducts to form high-quality films. They dissolved 1mmol of BiBr_3_ powder in 1.5mL DMSO, stirred continuously for 2h, added ethanol and centrifuged to obtain BiBr_3_(DMSO)_3_ powder. In addition, BiBr_3_(DMSO)_3_ is dried in a vacuum oven at 60 °C, so that the DMSO solvent evaporates to produce a milky yellow BiBr_3_(DMSO)_2_. Then CsBr, AgBr, and BiBr_3_(DMSO)_2_ were added to DMSO at the molar ratio of 2:1:1 and stirred for 2h, and the Cs_2_AgBiBr_6_ precursor solution was rotated on the substrate at 3000 rpm to form a film [53]. The BiBr_3_(DMSO)_2_ adduct was found to be a compound, not a mixture of BiBr_3_ and DMSO. The addition of adducts delays the rapid crystallization process and makes it easier to form larger grains. This also indicates that the formed film has high coverage and high smoothness. It provides a good idea and hope for the research of Cs_2_AgBiBr_6_. 

### 3.2. Vacuum Deposition 

The vapor deposition method needs to be carried out in a vacuum environment, so the requirements for the equipment are greater. Vapor deposition is a common method for growing thin films. However, for thin films of double perovskite materials, the exploration process is relatively slow because of a variety of material components and relatively complex technology.

In 2018, Wang et al. prepared Cs_2_AgBiBr_6_ thin films by sequential vapor deposition for the first time. AgBr, BiBr_3_, and CsBr are deposited on the substrate in sequence, and the diffusion reaction is driven by the post-annealing process, so the deposition sequence is important. More interestingly, BiBr_3_ is beneficial to the growth of crystals and the elimination of impurity phases, and the molar ratio of BiBr_3_:AgBr:CsBr is optimized from 1:1:2 to 1.5:1:2 to prepare a high-integrity film. This process is carried out in a vacuum environment, and the substrate support is placed approximately 27 cm above the evaporation source with a substrate rotation rate of 9 r/min. Wang et al. found that sequential vapor deposition requires deposition at a pressure less than 1 × 10^−3^ Pa, and CsCl, BiCl_3_, and AgCl are deposited in sequence [66]. In one cycle, the layer thickness can reach 110 nm. After annealing at 180 °C, a completely pure Cs_2_AgBiCl_6_ film with directional crystallization characteristics was obtained. Although the process is slightly more troublesome and takes longer than the spin-coating method, the phase purity and strength are superior.

In 2021, the workers formed Cs_2_AgBiBr_6_ powder by ball milling method and proposed pulsed laser deposition to form the film [67]. The powder was ground under a uniaxial pressure of 600 MPa and loaded into a disk-shaped target. It was then deposited by laser ablation in an argon environment. At 200 °C, a complete film with a thickness of 200–300 nm is formed. This method can realize near stoichiometric transfer for multicomponent compounds, which is conducive to the study of promoting the growth of thin films. 

For the multicomponent compound Cs_2_Ag_x_Na_1−x_BiyIn_1−y_Cl_6_, Oleksandr Stroyuk placed the powder in a crucible and heated the evaporation temperature to about 670 °C when the extraction pressure was 8 × 10^−6^ mbar, which is used by single-source vacuum deposition [68]. Until there is no powder in the crucible, the film deposition is finished, and the thickness of the film is about 250–300 nm. The phase and composition of the film are effectively controlled. These methods can control the growth of the multicomponent phase by adjusting the pressure and temperature and have a good demonstration for the formation of a new multicomponent system.

As a conclusion, the method of forming thin films by chemical solution deposition is more popular than the vapor deposition method, mainly because it is more convenient and the experimental conditions are simpler. However, the quality of the film and the regulatory chemical composition are better by vapor deposition. Therefore, for the chemical solution deposition method, finer process conditions under the premise of saving time and cost are attempted to achieve a more uniform and smooth film with high coverage. Some challenges, like the uneven transport layer film, the impurities floating on the surface, the different preparation environment of the transport layer and the absorbent layer, and the heating during the annealing process after spin-coating, need to be further solved. For the vapor deposition method, it is necessary to ensure the quality of the film while simplifying the process as much as possible. With the development of perovskite research, large-scale preparation has gradually entered people’s field of vision. The most common spin-coating method relies on the rotational speed to distribute the solution to the base (≤ 1 cm^2^), and in the case of a large base, this is not easy to achieve. In hybrid perovskites, numerous film-making methods have been investigated, for example, spin-coating, soft-cover deposition, brush painting, screen printing, slodie coating, and spray coating, etc. However, the research time of Cs_2_B′B″X_6_ double perovskite is still relatively short. At present, it mainly focuses on blade coating for large areas [69]. The main process drops the precursor solution on the substrate, quickly scrapes it with a glass blade to form a wet film, and then anneals to obtain a pure-phase film with an area of 10 cm^2^.

## 4. Performance Enhancement of Cs_2_B′B″X_6_ Double Perovskite Solar Cells 

At present, a variety of synthesis methods and thin-film manufacturing processes provide a solid foundation for the fabrication of devices. However, due to the low solubility of double perovskite precursors in organic solvents and the high temperature required for the preparation of pure-phase films, the film-forming quality is still not ideal and needs to be further improved. Moreover, the surface in contact with the perovskite layer also affects the development of photoelectric properties due to defects or the misalignment of energy bands between the two layers. The band gap of double perovskites, especially the most studied Cs_2_AgBiBr_6_, is large and indirect, which leads to high energy loss and limited light absorption capacity in photovoltaic device applications [70]. Therefore, how to reasonably optimize the process and improve performance is another major research focus. Among many photovoltaic devices, the research of new energy solar cells has always attracted attention. In this chapter, we make three strategies for optimizing solar cells, which are film quality optimization, band gap adjustment, and interface engineering.

### 4.1. Film Quality Optimization

The quality of the absorbent layer directly determines the device performance of the whole thin-film solar cell. Smooth film morphology, uniform composition, pure phase, and good crystallization are essential for the fabrication of high-performance solar cell devices. When using the spin-coating method to form a thin film, the quality of the thin film will be affected by the preheating temperature, the experimental environment, the spin-coating speed, the spin-coating duration, the annealing temperature, and the addition of an anti-solvent [71,72,73,74]. Doping or film modification in the film material can also optimize the film quality [75,76]. From the film manufacturing methods in the previous chapter, it can be seen that chemical solution deposition is often used. Therefore, this chapter mainly focuses on optimizing thin films prepared by chemical solution deposition.

#### 4.1.1. Preheating

**Figure 3:**
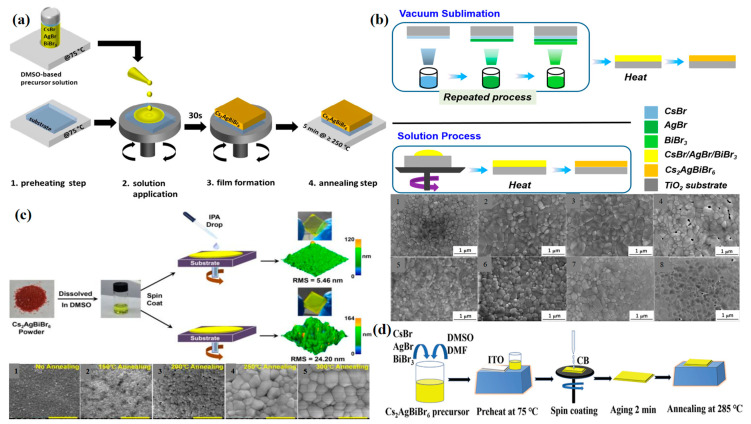
(**a**) Schematic of the synthesis route for Cs_2_AgBiBr_6_ thin films (reproduced with permission [64], 2017, Royal Society of Chemistry); (**b**) schematic representation and SEM about the preparation of Cs_2_AgBiBr_6_ thin films by solution-processing (annealed at (1) 240 °C, (2) 260 °C, (3) 280 °C, and (4) 300 °C) and vacuum-sublimation (annealed at (5) 180 °C, (6) 200 °C, (7) 220 °C, and (8) 240 °C) (reproduced with permission [33], 2019, American Chemical Society); (**c**) schematic illustration of the spin-coating process with and without anti-solvent dropping protocol, and SEM of Cs_2_AgBiBr_6_ films annealed under different temperatures ((1) no annealing at (2) 150 °C, (2) 200 °C, (3) 250 °C, and (4) 300 °C) (reproduced with permission [21], 2018, John Wiley and Sons); (**d**) deposition process of the Cs_2_AgBiBr_6_ thin film (reproduced with permission [77], 2020, Springer).

The preheating of the precursor solution and the substrate before coating has an effect on the crystallization rate. When the preheating temperature changes, the solvent in the solution will evaporate quickly, which is conducive to increasing the nucleation density (Figure 3a). Greul and his group fabricated FTO/c-TiO_2_/mp-TiO_2_/Cs_2_AgBiBr_6_/Spiro-OMeTAD/Au devices. The research hypothesis shows that the performance is best at the preheating temperature of 75 °C. If the preheating condition reaches 100 °C, the top layer of large crystals will be formed on the TiO_2_ mesoporous layer, which is difficult to transfer electrons due to the thickness of the layer [64].

Under the optimal preheating temperature, the agglomeration phenomenon on the surface of the mesoporous layer is reduced, which also shows the uniform morphology and the optimized photoelectric performance. Zhao et al. also preheated the film before preparation to promote solvent evaporation, thereby increasing the nucleation density [73]. However, there will also be special conditions; one situation is that if the preheating step is not carried out, the perovskite crystal strength is slightly reduced, resulting in at incomplete coverage of the film and lower film quality, which further leads to a reduction in the light absorption efficiency, which means that the current of the device is reduced. The second situation is that if only the solution is preheated, when the hot solution is added to the cool substrate, the crystal nucleation rate and the film quality will be correspondingly worse. The preheating step is used in most experimental processes at present.

Therefore, the substrate and precursor solution are heated before spin-coating so that the solution continues to be fully dissolved, and since the substrate temperature is the same as the solution temperature during spin-coating, the solution will not be cooled. Under the spin-coating process, the appropriate preheating temperature can promote crystallization and the formation of a uniform film.

#### 4.1.2. Post-Annealing

Besides preheating, another method of controlling the temperature of the process is post-annealing. The post-annealing process is an essential step for films prepared by chemical solution deposition. The reason is that the increase in temperature promotes the evaporation of solvent, eliminates residual solvent and other volatile components, which is conducive to an improvement in membrane integrity, and promotes the generation of pure phase. In addition, nucleation is encouraged during the annealing process, which is conducive to increasing the grain size and optimizing the crystallinity of the film.

In 2016, Xiao et al. predicted using DFT calculations that the potential chemical region of Cs_2_AgBiBr_6_ is narrow and side phases (such as Cs_3_Bi_2_Br_9_) are easily generated during synthesis [74], which will affect the photoelectric properties of double perovskites. Therefore, the choice of annealing temperature should be taken seriously. Greul et al. experimentally eliminated the side phase and studied the annealing temperature conditions [60] and found that the required double perovskite phase could be formed only when the annealing temperature was at least 250 °C with chemical solution deposition. When the annealing temperature reaches 250 °C, the pure phase is formed; 250 °C is the lower limit for complete conversion to the pure phase of double perovskite. But at 300 °C, double perovskite will fall off the substrate, and its performance will weaken. And at a lower temperature of 150 °C, the metal–solvent intermediate can be removed, but the side phase is not eliminated. Moreover, McClure et al. demonstrated the formation of Cs_2_AgBiBr_6_ in a solid-state reaction at 210 °C, which is also close to the temperature at which the pure phase is obtained [34].

Comparing the annealing temperatures of films treated with vacuum and solution, the authors found that the films treated by vacuum are more likely to form heat-induced diffusion reactions under certain conditions [33]. The annealing temperatures of the vacuum-treated films were 180 °C, 200 °C, 220 °C, and 240 °C. In order to improve the crystallinity of the films, the annealing time was set at 30 min (Figure 3b). The annealing temperatures of the films treated with solution spin-coating were 240 °C, 260 °C, 280 °C. and 300 °C, and the annealing time was set as 5 min. The peak intensity of both methods increases with the increase in the annealing temperature. Through the characterization analysis, it was found that the peak strength of the vacuum-treated film was 220 °C, the solution-treated film was 280 °C, and the crystallinity of the solution-treated film was higher than that of the solution-treated film. Compared with the vacuum-treated film, the solution-treated film has higher crystallinity, a lower band gap, a longer carrier lifetime, and higher mobility.

Therefore, after the spin-coating process, the choice of the annealing temperature is very particular. The purpose of annealing is to form a pure-phase substance and eliminate excess material. Annealing at the appropriate annealing temperature can generate a pure-phase material, which also promotes the continuous growth of grains and an increase in size, further improving the integrity of the film.

#### 4.1.3. The Choice of Anti-Solvent 

The method of adding an anti-solvent to lead-free double perovskite was borrowed from the manufacturing of hybrid perovskite. Its main function is to accelerate the solvent extraction in the spin-coating process of perovskite, improve the nucleation density, promote a smooth surface, and improve the quality of the film. Therefore, the process of adding anti-solvent is more conducive to the formation of the film and improving the photoelectric performance. Therefore, in the selection of an anti-solvent, researchers added toluene, chlorobenzene, methanol, ethanol, and isopropanol, and only the IPA solvent film formed the best in a scanning electron microscope (SEM) comparison in Figure 3c. As the Cs_2_AgBiBr_6_ material is an inorganic compound, IPA can effectively remove organic matter on the surface of the material, and the addition of an anti-solvent can promote the crystallization of the film, so the addition of an IPA anti-solvent can effectively form a smooth, non-porous, and large grain-size film.

By comparing the presence or absence of an anti-solvent, the average root roughness of the films without IPA is four times that after the addition of IPA, the appearance of the films is frosted and granular, and there are obvious holes. The surface of an IPA film is bright and smooth, and the quality is obviously improved when using an isopropyl alcohol (IPA) anti-solvent to optimize the film quality and adjusting the annealing temperature to form a film with a uniform and smooth surface [21].

In addition to the choice of the anti-solvent, because the anti-solvent is closely related to the solvent, the selection of organic solvents in perovskite precursors is also necessary. In the preparation of a precursor solution, Gao et al. studied the best solvent for dissolving Cs_2_AgBiBr_6_ powder. Through the comparison of four polar solvents, DMF, DMSO, γ-butyralactone (GBL), and N-accelerated pyrrolidone (NMP), it was found that the powder was only completely dissolved in 0.4 M DMSO. The other three solvents barely dissolved the powder [77,78,79].

However, other researchers mixed the two solvents of different proportions in perovskite precursor solutions. The solubility of the DMSO precursor is good, but the high boiling point (189 °C) and low vapor pressure (0.42 mmHg at 20 °C) are not ideal solvents. DMF has a lower boiling point (153 °C) and a higher vapor pressure (2.7 mmHg at 20 °C), which is more suitable for grain growth and high-quality film formation [80]. Zhao et al. dissolved 0.8 mmol of CsBr, 0.4 mmol of AgBr, and 0.4 mmol of BiBr_3_ in a DMSO and DMF mixture (*v*:*v* = 1:1) and stirred for several hours at 60–80 °C until complete dissolution (Figure 3d). The precursor solution and the substrate were preheated at 75 °C. And 10 s before the end of dynamic spin-coating (at the gelation point), Chlorobenzene (CB) was added to the anti-solvent. After spin-coating, it was left to stand for 2 min, then placed on the hot table and annealed at 285 °C for 5 min.

However, when the volume ratio of DMSO to DMF is less than 1, the solubility of Cs_2_AgBiBr_6_ decreases, so a volume ratio of 1 is the most suitable. According to the Ostwald ripening process, aging and post-annealing processes are the keys to grain growth. In the post-annealing process, the addition of an anti-solvent also promotes the evaporation of the solvent, reduces the size and density of the pinhole, and accelerates the nucleation density of the coating.

Therefore, on the one hand, when dissolving the solute, the choice of solvent is also very critical, and the solvent suitable for the polarity and coordination degree of double perovskite materials should be selected, which is conducive to the film-forming power and quality. On the other hand, in the process of spin-coating, the process of dropping an anti-solvent can accelerate the nucleation of crystals and increase the nucleation density. For Cs_2_AgBiBr_6_ materials, the addition of an IPA solvent can effectively remove the organic matter on the surface, so it can be seen that the roughness is reduced and a film with large grains is formed.

### 4.2. Band Gap Adjustment

In addition to the optimization of material synthesis and preparation, at the same time, the properties of the material itself are explored. Previous studies based on experiments and calculations have shown that many Cs_2_B′B″X_6_ double perovskite materials have direct band gaps and are relatively wide [81]. This results in the unsatisfactory performance of optoelectronic devices. Kangsabanik et al. have proposed that halide double perovskite materials (such as Cs_2_(Ag_0.75_Pb_0.25_) (Bi_0.75_Pb_0.25_)Br_6_) can be converted from an indirect to a direct band gap by the incorporation of small amounts of Pb^2+^ at the B′ and B″ positions [82]. Although first-principle calculations predict better performance than CH_3_NH_3_PbI_3_, the reason for toxicity makes this class of compounds not very attractive [83]. Therefore, the combination of good photophysical properties of halide double perovskites with lead-free toxicity and environmental stability has been promoted through theoretical and empirical studies of the photoelectric properties of the compounds [84]. The configuration of lead-free halide double perovskite is mainly the substitution of monovalent and trivalent cations to form A_2_B(Ⅰ)B(Ⅲ)X_6_ double perovskite. Therefore, one way to adjust the band gap is that, because the material structure belongs to the cubic crystal system, the original configuration of the material will be changed under the action of pressure or temperature, resulting in a change in the band gap. Another method is to change the atomic arrangement for structural transformation, by adding dopants to the double perovskite material to change the band gap [85,86,87]. This section will focus on the methods of structural transition and functional doping to adjust the band gap.

#### 4.2.1. Structural Transition

**Figure 4:**
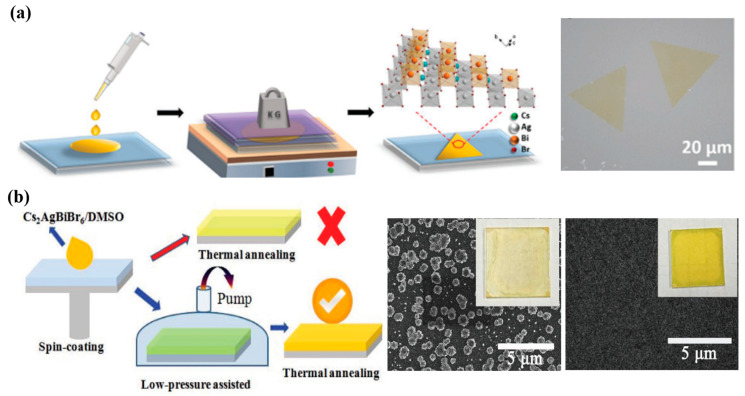
Double perovskite thin films by pressure treatment. (**a**) Schematic illustration of 2D Cs_2_AgBiBr_6_ grown by space-confined method and SEM image (reproduced with permission [58], 2021, John Wiley and Sons); (**b**) the Cs_2_AgBiBr_6_ film fabrication process diagram, and SEM images of film obtained by TA and LPA process (reproduced with permission [14], 2018, John Wiley and Sons).

Common double perovskite materials are three-dimensional materials, and their transmission capacity is not as good as that of two-dimensional materials. Therefore, the direct preparation of two-dimensional double perovskite materials is also a good attempt [11]. Figure 4a shows that CsBr, BiBr_3_, and AgBr were added to the HBr solution at a molar ratio of 2:1:1 and stirred at 120 °C for 2 h to obtain the precursor solution [58]. The precursor solution was gradually dripped onto a clean sapphire substrate, then heated at 70 °C for 24 h and cooled to room temperature to ensure good contact between the substrate and the sample. To obtain 2D Cs_2_AgBiBr_6_, a pressure of 50 KPa was applied to the whole body during heating for 24 h, which could be achieved by using weight to further limit the growth space. The two substrates were then separated to obtain bulk Cs_2_AgBiBr_6_ on the sapphire substrate. The ultra-thin 2D Cs_2_AgBiBr_6_ has a shorter lifetime and produces more free carriers at the edge. 2D Cs_2_AgBiBr_6_ nanosheets exhibit significant advantages over bulk nanosheets in light detection because they promote light–matter interaction. By controlling the growth space and the diffusion of solution on the substrate, the Cs_2_AgBiBr_6_ preparation from bulk to the two-dimensional limit has been achieved, which provides prospects for the growth of perovskite semiconductors in the optoelectronic field.

Another interesting pressurization method is the preparation of high-quality, stable double perovskite Cs_2_AgBiBr_6_ films by a low-pressure assisted (LPA) method, as shown in Figure 4b. In this method, with the assistance of low pressure, the material nucleates, grows, and forms a continuous film with high integrity. It is the first lead-free double perovskite planar heterojunction solar cell with a high-quality Cs_2_AgBiBr_6_ film, and the PCE reaches 1.44%. Firstly, CsBr, AgBr, BiBr_3_, and HBr were dissolved at 110 °C and stirred for 120 min [14]. The solution was cooled to room temperature overnight. In this synthesis method, the previously synthesized Cs_2_AgBiBr_6_ powder was dissolved in the DMSO solvent to form a yellowish precursor solution of 0.5 mol L^−1^, which was coated on the substrate by rotating at 2000 rpm. Then the film was quickly moved to a low-pressure chamber where the pressure reached 20 Pa, and the film’s color gradually changed from transparent to light yellow. Then, in order to make the residual solvent evaporate, the film was annealed at 200 °C to obtain a smooth and uniform film with good crystallization. Compared with the traditional annealing method, it is less easy to obtain a thin film of island particles.

**Figure 5:**
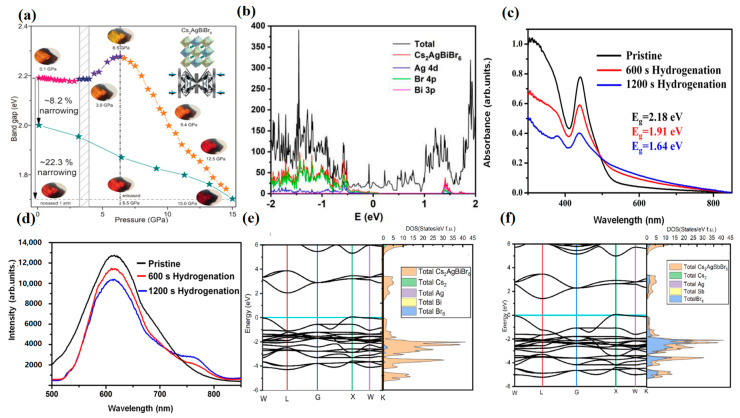
(**a**) Band gap evolution of Cs_2_AgBiBr_6_ crystal at high pressure (reproduced with permission and the representative optical micrographs showing piezochromic transitions [28], 2017, John Wiley and Sons); (**b**) partial density of states (PDOS) of Cs_2_AgBiBr_6_ (001) @Ti_3_C_2_O_2_ (reproduced with permission [90], 2022, Elsevier); (**c**) Ultraviolet–visible absorption spectra (UV–vis) and (**d**) PL spectra of the Cs_2_AgBiBr_6_ films with different hydrogenation time (reproduced with permission [92], 2022, Springer Nature); band structures and density of states of (**e**) Cs_2_AgBiBr_6_, and (**f**) the band gap and DOS of Cs_2_AgSbBr_6_ (reproduced with permission [93], 2022, Elsevier).

In addition to the direct formation of two-dimensional double perovskite materials, the three-dimensional material structure can also be transformed by pressure or heating. Some researchers have used first-principles calculations to predict that, under pressure or heating, a material will undergo an order–disorder transition, resulting in an indirect band gap becoming a direct band gap. In 2017, Li et al. pressurized Cs_2_AgBiBr_6_ from 0 to 15 GPa and found that the pressure changed the lattice and that there were phase transitions and amorphous states, which could lead to a smaller band gap (Figure 5a) [28]. At 15 GPa, the band gap reaches the minimum value of 1.7 eV, while during the pressurization process, the trend is first rising and then decreasing, and different pressure values have different structural changes, mainly due to the change in octahedron and bond length in the double perovskite. A change in temperature can have a similar effect. Zhang et al. used the thermochromic principle to anneal the synthesized single crystal at 400 °C, and Ag and Bi atoms randomly occupied the B position, forming a disordered Cs_2_AgBiBr_6_ structure [88]. The formation of the reverse defect makes the band edge wider and the band gap smaller.

#### 4.2.2. Functional Doping

With another method for decreasing the band gap, workers target the reasons that limit performance and solve the problem by doping, such as metal and organic materials. Compared with MAPbI_3_ [89], Cs_2_AgBiBr_6_ has a large band gap and a weak light absorption capacity. Therefore, Yang et al. added Ti_3_C_2_T_x_ MXene to improve the performance of dye-sensitized double perovskite solar cells [90]. From the DOS curve, it can be seen from Figure 5b that the valence band is mainly contributed by the 4p orbital of Br and the 4d orbital of Ag, while the conduction band is mainly contributed by the 3p orbital of Bi and the 4p orbital of Br. Experiments and calculations show that the Cs_2_AgBiBr_6_ (001) surface is the most stable structure. For Cs_2_AgBiBr_6_ doped with Ti_3_C_2_O_2_, the band gap at (001) is the same as that of undoped Cs_2_AgBiBr_6_. The purpose of adding D149 dye is to enhance and broaden the light absorption capacity, but also because D149 can photogenerate charge carriers, there will be a non-radiative recombination loss between D149 and Cs_2_AgBiBr_6_, resulting in a decrease in the open circuit voltage in the solar cell. However, the doping of Ti_3_C_2_T_x_ with Cs_2_AgBiBr_6_ (001) affects the intrinsic material; the valence band moves up after doping; and the weak van der Waals interaction between Cs_2_AgBiBr_6_(001)@Ti_3_C_2_T_x_ retains the semiconductor properties. In addition, the author fabricated the device and found from the experimental results that the photocurrent was significantly improved, and the final efficiency reached 4.47%. This also proves that the addition of Ti_3_C_2_T_x_ with a high power function effectively improves performance [91].

Using Cs_2_AgBiBr_6_ as a photovoltaic cell formed by a perovskite layer, Zhang et al. obtained the current highest device efficiency through hydrogenation. Firstly, Cs_2_AgBiBr_6_ films of high quality were prepared by the traditional one-step spin-coating method [92]. The films were yellow films with a face-centered cubic phase. The perovskite film was then hydrogenated at different times (600 s and 1200 s) using plasma treatment in a mixture of a hydrogen (H_2_) and argon (Ar) gas environment. With the continuous increase in hydrogenation time, the optical absorption of wavelengths above 496 nm increases significantly, which indicates that the visible light absorption of Cs_2_AgBiBr_6_ perovskite film is effectively improved during 0–1200 s hydrogenation. Moreover, the intensity of the photoluminescence (PL) peak at about 760 nm gradually increases with the increase in time, which also indicates that the band gap of Cs_2_AgBiBr_6_ gradually decreases (Figure 5c,d). Previous research groups suggested that the formation of defects between the valence band and conduction band leads to enhanced sub-band gap absorption but also confirmed a decrease in PL strength and carrier lifetime [94,95]. 

When the hydrogenation time is extended to 1200 s, the carrier mobility is increased by 542%, and the carrier concentration is increased by about four times. However, the carrier lifetime does not shorten, which also proves that the band gap of the Cs_2_AgBiBr_6_ film does decrease after hydrogenation, but it is not caused by the increase in defect density in the perovskite absorption layer. Moreover, the ITO/SnO_2_/Cs_2_AgBiBr_6_/Spiro-OMeTAD/Au structure device is fabricated to ensure the compatibility of each level in the device. In the measurement results, the Voc increased slightly, but the Jsc increased from 1.03 mA cm^−2^ at the beginning to 7.65 mA cm^−2^ after 1200 s hydrogenation. Moreover, the PCE value obtained by reverse scanning reached 6.37%, which was the highest efficiency record of a Cs_2_AgBiBr_6_ solar cell so far.

In a theoretical calculation, the density of states (DOS) can effectively show the main contribution positions of elements [96]. Therefore, for the most commonly studied Cs_2_AgBiBr_6_, the Br element mainly contributes to the valence band, while the Bi element mainly contributes to the conduction band [97]. This is an important reference for the doping optimization of material properties. Therefore, anions and ions on two heterovalent substituents have been extensively studied in the experiment.

For example, the band gap of Cs_2_AgBiBr_6_ and Cs_2_AgSbBr_6_ was searched by Mukhtar [93]. The band structure of the double perovskite shows an indirect band gap in the L-X symmetric direction, where the X position is the maximum valence band and the L position is the minimum conduction band (Figure 5e,f). The calculated band gaps of Cs_2_AgBiBr_6_ and Cs_2_AgSbBr_6_ are 2 eV and 1.39 eV, respectively. With the increase in atomic radius, the influence of electrostatic force on valence shell electrons decreases, so that the band energy between the valence band and conduction band decreases. The DOS showed that Bi (6p) or Sb (5p) and Br (4p) antibonding formed double perovskite compounds. When Li et al. studied Cs_2_Ag(Sb_x_Bi_1−x_)Br_6_, the researchers demonstrated a type II band arrangement with a valence band offset (VBO) of 0.75 eV and a conduction band offset (CBO) of 0.34 eV. When Bi is added to pure Sb double perovskite, a “Bi-like” low-energy conduction band state is generated due to the preferential assignment of CBM wave function amplitude to Bi sites, and the conduction band decreases. Bi elements also contribute to spin-orbit coupling, leading to a decrease in conduction band energy. Not only that, the highest energy valence band state will remain “Sb-like” and generate large wave function amplitudes at the Sb site, with the valance band maximum (VBM) only slightly lower than pure Sb material. The mixed electron states in the alloy produce a low energy conduction band dominated by the Bi–Br interaction and a high energy valence band dominated by the Sb–Ag–Br interaction, resulting in a lower band gap of the double perovskite alloy than the two pure materials.

### 4.3. Interface Engineering

#### 4.3.1. Band Gap Alignment

The interface between the electron/hole transport layer and the perovskite layer in PSCs is very important for carrier extraction and transport. Surface defects may be caused by the manufacturing processes of thin films and interlayer forming, which affect the separation of photogenerated carriers into free charges at the boundary of the absorption layer and the transport layer and form non-radiative recombination at the interface, thus leading to the performance of PSCs. Moreover, if the bands between layers do not match, electrons and holes cannot be effectively transported, and the recombination phenomenon may occur, so it is necessary to adjust the band alignment. Therefore, the interface engineering method is very important to improve the performance of solar cells. Two strategies, interfacial energy level adjustment and surface defect passivation, have been used to optimize solar cell performance to improve PCE [98]. Band alignment is a fundamental element of solar cell structure. For the perovskite layer and electron transport layer, it is found that the electron diffusion length is smaller than the hole length by PL and other tests, which leads to poor carrier extraction in the device. Zhao et al. studied the method of constructing bulk heterojunctions in Cs_2_AgBiBr_6_ PSCs with the TiO_2_ mesoporous layer to increase the short-circuit current density. They studied the diffusion rate and photo-generated carrier strength of different transport materials, mainly Spiro-OMeTAD and PCBM as hole transport materials and TiO_2_ mesoporous layers in the electron transport layer. It is important that the ITO/c-TiO_2_/mp-TiO_2_/Cs_2_AgBiBr_6_/Spiro-OMeTAD/Au structure has a higher efficiency compared to the ITO/c-TiO_2_/Cs_2_AgBiBr_6_/Spiro-OMeTAD/Au structure because the newly added electron transport layer has a mesoporous structure, which can extract more electrons and transport electrons faster [99].

For the hole transport layer, the energy levels of lead-free double perovskites, such as Spiro-OMeTAD and P_3_HT, are not matched very well. Moreover, organic hole transport materials are usually expensive and have poor stability, which is also one of the problems for the low efficiency of Cs_2_B′B″X_6_ double perovskite. Xiao et al. found that using Cu_2_O, an inorganic material, as a hole transport layer improved the device’s performance. Cu_2_O has a direct band gap with a band gap width of 2.1 eV. The authors used the vacuum deposition method to make a film thickness of 50 nm and obtained the optimal device with an improved efficiency of 1.52% and high stability [59]. Other workers also searched for appropriate hole transport materials. Pang et al. reported the use of MoS_2_ nanosheets as hole transport interlayers to improve device performance. Due to the high carrier mobility and chemical stability of MoS_2_ and the suitable energy levels to match the perovskite layer, the PCE reaches as high as 4.17% [100]. Nowadays, some solar cell software has emerged with good predictions in terms of structure and performance. For example, the selection of the appropriate electron transport layer according to band alignment predicts the performance of the new battery structure, which provides reliable support for the improvement in performance in the experiment [101,102,103].

#### 4.3.2. Surface Passivation

Surface passivation is the key to effectively reducing carrier recombination and improving carrier lifetime because efection-induced recombination on the surface of Cs_2_AgBiBr_6_ films is faster than defection-induced recombination in bulk films. Wang et al. introduced a carboxy-containing chlorophyll derivative carboxy-Chl (C-Chl) as a photosensitizer to desensitize the mesoporous TiO_2_ material and used it as an electron transport layer in perovskite solar cells, which was the first time that dye-sensitized perovskite solar cells were prepared. The device with the FTO/c-TiO_2_/mp-TiO_2_/C-Chl/Cs_2_AgBiBr_6_/Spiro-OMeTAD/Ag structure was prepared. The study found that C-Chl helps to improve the light absorption capacity in the visible range, and there is an absorption peak near 680 nm, increasing the light absorption range and ultimately reaching an energy conversion efficiency of 3.11% [104]. 

Another unique dye has been developed for solar cells. Yang et al. introduced N719 dye between the hole transport layer and the perovskite layer. The dye can broaden the optical absorption spectrum, reduce the surface defects of Cs_2_AgBiBr_6_ film, and accelerate carrier extraction, with the final PCE as high as 2.84% [105]. These studies not only contribute to reducing defects and speeding up carrier extraction and transport but also provide an efficient and convenient method to improve the photoelectric efficiency and stability of PSCs. 

Defects are mainly produced in the process of film crystallization, including point defects and extended defects, which can be divided into shallow-level defects and deep-level defects. Many types of defects are the main cause of severe non-radiative recombination within the device, which in turn limits the improvement in photovoltaic characteristics and stability of solar cell devices. Nowadays, organic molecules have become effective additives in surface passivation because of the function of functional groups and bond connections. Although the passivation work is becoming more and more mature, the type, concentration, and trap depth of defects cannot be accurately pointed out, so the experimental characterization and passivation mechanism need to be further explored.

Although many optimization methods have been developed, the application of double perovskite in solar cells still lags far behind that of hybrid perovskite. The most studied Cs_2_AgBiBr_6_ lead-free double perovskite has a maximum efficiency of 6.37% so far, which is also severely limited because of its wide band gap. And other lead-free double perovskite materials, Cs_2_B′B″X_6_, in the material synthesis are still under constant exploration, but in terms of the current realization of materials, the development space is huge, and the future in the application field, not only in the direction of solar cells but also in detectors, sensors, catalysis, and other fields, has great potential.

## 5. Summary and Outlook

The Cs_2_B′B″X_6_ material has the advantages of good stability, being friendly to the environment, having an adjustable band gap, and having a high light absorption coefficient. And the material characteristics have well resolved the lead toxicity and instability problems of hybrid perovskite, which quickly entered the photovoltaic application to become a strong competitor. However, due to the diversity of elements and solubility, the synthesis method is more complicated than that of hybrid perovskite. In order to prepare materials with high phase purity, solid-state synthesis and solution-based synthesis methods are generally selected. Solution-based synthesis methods are more extensive, less costly, and more economical in experiment time, while solid-state synthesis methods form crystals that are purer and more environmentally friendly. Moreover, the method for preparing thin film can be divided into vacuum deposition and chemical solution deposition. And many methods have been derived from lead-based perovskites to manipulate and control film growth. Nowadays, spin-coating is the most widely used method for preparing thin films because it is convenient and easy to operate. However, for mass synthetic material, as well as large-scale production of thin films for commercial applications, how to prepare and retain the photoelectric properties of the material is our long-term goal, which needs more research.

For photovoltaic applications, the investigation of solar cell devices, reducing defects, and speeding up carrier separation and transport to improve performance are top priorities. Therefore, three strategies of film optimization, band gap regulation, and surface engineering are proposed. First of all, the optimization of the process is to improve the material’s crystallization, reduce the generation of defects, and thus can form a more complete film. Proper temperature, pressure, and the addition of solvents are conducive to the formation of high-quality films. Secondly, the perovskite band gap suitable for solar cells is about 1.4 eV, and materials with the ideal band gap can generate more photogenerated carriers. Compared to pressure or temperature regulation, functional doping is the most common method. It can be adjusted for the material contribution and changed for the electronic structure, which can obtain the promising material. As a result, defects and side phases problems may arise. Finally, the heterojunction structure is formed between the absorption layer and the transport layer for band alignment, which is conducive to the transport of electron holes and can effectively reduce non-radiative recombination. The other method is surface passivation, which is used to reduce surface defects, promote carrier transmission, and improve the photoelectric conversion efficiency of the device through additives such as effective functional groups. On the other hand, the promising Cs_2_B′B″X_6_ double perovskite material could be used in other photovoltaic devices. For example, Cs_2_AgBiBr_6_ with good stability is used in photocatalysis, and Cs_2_AgBiCl_6_ single crystals with high sensitivity are used in UV detectors. With limited compound availability, it is possible to find good materials to match photovoltaic devices with different characteristics.

Therefore, based on more and more research on Cs_2_B′B″X_6_ double perovskite materials and with more and more mature technology, there are still some strategies to explore. First of all, in the material synthesis, the synthesis methods of lead-free double perovskite crystals are various and have gradually applicated, and in the direct precursor solution, polar solvents should continue to be found to further solve the solubility problem. Secondly, in the film preparation, the film thickness of the simple and convenient spinning coating method is inconsistent, and the adhesion of the surface of the transmission layer needs to be further optimized. In solar cell applications, the band gap regulation of lead-free double perovskite is directly related to the light absorption capacity of solar cells, and the ideal band gap of perovskite is about 1.3–1.5 eV, so a certain proportion of doped materials with small band gaps can be doped to neutralize or develop new element materials. In addition, suspension bonds on the surface of the light-absorbing layer may be eliminated by ions or organics, and the characterization and mechanism of surface defects need to be further explored. Moreover, compared with traditional perovskites, Cs_2_B′B″X_6_ double perovskite has a large and indirect band gap, and the carrier transport rate from the light-absorbing layer to the transport layer is low, resulting in unsatisfactory power conversion efficiency. And after the band gap adjustment, the defects caused by the synthesis of the material cannot be balanced. There may be hope for this from theoretical simulations.

On the other hand, finding new lead-free double perovskite materials is very necessary for promoting the progress of perovskite. Machine learning and computational methods can be used to predict the possible existence of new materials; conditions like tolerance factors, decomposition conditions, and mechanical stability need to be considered; and the probable performance is obtained by calculating the band gap. In band alignment, the continuous search for materials that can be aligned with the absorption layer and the cost of the appropriate transport layer band to form a continuous heterojunction, especially the choice of the light absorption layer and the hole transport layer, the common spiro-OMeTAD/C transport hole is not perfectly matched, so the search for hole transport layer materials should continue. And it can be simulated using simulation software to prepare for feasibility in the experiment. Overall, Cs_2_B′B″X_6_ double perovskite provides a new pathway to achieve high efficiency and stability in perovskites. Finally, we believe in and demonstrate the promise of lead-free double perovskite materials in solar cells and other photovoltaic device applications.

## Figures and Tables

**Table 2:** Summary of film preparation conditions of Cs_2_B′B″X_6_ double perovskite compounds.

Compound	Synthesis Method	Synthesis Atmosphere	Heating Condition	Film Thickness	Grain Size	Year and Reference
Cs_2_Ag(Sb_x_Bi_1−x_)Br_6_	spin-coating	N_2_	preheating at 75 °C, post-annealing 135 °C for 5 min	200 nm	80 nm	2020 [26]
Cs_2_AgSbBr_6_	spin-coating		preheating at 200 °C, post-annealing 150 °C for 30 min			2019 [46]
Cs_2_AgBiBr_6_	spin-coating		preheating at 75 °C, post-annealing 285 °C for 30 min			2019 [46]
Cs_2_AgBiBr_6_	spin-coating		preheating at 100 °C, post-annealing 200 °C for 5 min	150 nm		2018 [50]
2D Cs_2_AgBiBr_6_	space-confined	a pressure of 50 kPa	70 °C for 24 h	9.8 nm	10μm	2021 [58]
Cs_2_AgBiBr_6_	sequential vapor deposition		post-annealing 250 °C for 30 min	120 nm AgBr, 200 nm BiBr_3_, 200 nm CsBr	500 nm	2021 [59]
Cs_2_AgBiBr_6_	spin-coating	glovebox	post-annealing 250 °C for 10 min		800 nm	2021 [60]
Cs_2_AgBiBr_6_	spin-coating		post-annealing 280 °C for 5 min		500 nm	2021 [47]
Cs_2_AgBiBr_6_	spin-coating	Ar	preheating at 75 °C, post-annealing 285 °C for 5 min	200 nm	28/34 nm	2021 [61]

## Data Availability

No new data were created or analyzed in this study. Data sharing is not applicable to this article.
